# Module structure of interphotoreceptor retinoid-binding protein (IRBP) may provide bases for its complex role in the visual cycle – *structure/function study of Xenopus IRBP*

**DOI:** 10.1186/1471-2091-8-15

**Published:** 2007-08-04

**Authors:** Federico Gonzalez-Fernandez, Claxton A Baer, Debashis Ghosh

**Affiliations:** 1 Ross Eye Institute, Departments of Ophthalmology and Pathology, State University of New York, Medical Research Service, Veterans Affairs Medical Center, Buffalo, New York, USA; 2 Duke University Eye Center, Durham, North Carolina, USA; 3 Hauptman-Woodward Institute, Department of Pharmacology and Therapeutics, Roswell Park Cancer Institute, USA; 4 Department of Structural Biology, State University of New York, Buffalo, USA

## Abstract

**Background:**

Interphotoreceptor retinoid-binding protein's (IRBP) remarkable module structure may be critical to its role in mediating the transport of all-*trans and 11-cis *retinol, and 11-*cis *retinal between rods, cones, RPE and Müller cells during the visual cycle. We isolated cDNAs for *Xenopus *IRBP, and expressed and purified its individual modules, module combinations, and the full-length polypeptide. Binding of all-*trans *retinol, 11-cis retinal and 9-(9-anthroyloxy) stearic acid were characterized by fluorescence spectroscopy monitoring ligand-fluorescence enhancement, quenching of endogenous protein fluorescence, and energy transfer. Finally, the X-ray crystal structure of module-2 was used to predict the location of the ligand-binding sites, and compare their structures among modules using homology modeling.

**Results:**

The full-length *Xenopus *IRBP cDNA codes for a polypeptide of 1,197 amino acid residues beginning with a signal peptide followed by four homologous modules each ~300 amino acid residues in length. Modules 1 and 3 are more closely related to each other than either is to modules 2 and 4. Modules 1 and 4 are most similar to the N- and C-terminal modules of the two module IRBP of teleosts. Our data are consistent with the model that vertebrate IRBPs arose through two genetic duplication events, but that the middle two modules were lost during the evolution of the ray finned fish. The sequence of the expressed full-length IRBP was confirmed by liquid chromatography-tandem mass spectrometry. The recombinant full-length *Xenopus *IRBP bound all-*trans *retinol and 11-*cis *retinaldehyde at 3 to 4 sites with *K*_*d*_'s of 0.2 to 0.3 μM, and was active in protecting all-*trans *retinol from degradation. Module 2 showed selectivity for all-*trans *retinol over 11-cis retinaldehyde. The binding data are correlated to the results of docking of all-*trans*-retinol to the crystal structure of *Xenopus *module 2 suggesting two ligand-binding sites. However, homology modeling of modules 1, 3 and 4 indicate that both sites may not be available for binding of ligands in all four modules.

**Conclusion:**

Although its four modules are homologous and each capable of supporting ligand-binding activity, structural differences between their ligand-binding domains, and interactions between the modules themselves will be critical to understanding IRBP's complex role in the visual cycle.

## Background

The transport of retinoids and fatty acids between the retinal pigment epithelium (RPE) and retina is critical to photoreceptor development, structure and function. The exchange of visual cycle retinoids and possibly fatty acids between these two cell layers is mediated by interphotoreceptor retinoid-binding protein (IRBP) [reviewed in [1-5]]. IRBP is the most abundant protein component of the interphotoreceptor matrix, the extracellular material separating the RPE and retina. In contrast to most retinoid-binding proteins, which are present in a wide variety of tissues, IRBP is expressed uniquely by cells of photoreceptor origin [6-10].

Within the matrix IRBP may have several roles in mediating the transport of retinoids in the visual cycle. By binding retinoids, IRBP solubilizes all-*trans *retinol and 11-*cis *retinal while protecting these retinoids from isomeric and oxidative degradation [11]. IRBP also appears to support the removal of all-*trans *retinol from bleached outer segments [12], delivery of all-*trans *retinol from rods to the RPE [13], and the transfer of 11-*cis *retinal from the RPE to the rods [14-17]. Finally, IRBP may have an important although little-understood role in retinal development [1,18-20], and fatty acid trafficking [21-23].

IRBP is large (135 kDa in human) compared to other retinoid-binding proteins [24]. For example, serum retinol-binding protein is 21 kDa (183 amino acid residues). IRBP's size is partly due to the fact that its gene is composed of multiple homologous repeats. Each repeat codes for a module of ~300 amino acid residues. Mammalian and avian IRBPs are composed of 4 modules. In contrast, teleost IRBPs contain only two modules [25,26]. In all vertebrate classes examined to date, *IRBP's *three introns are located in the repeat coding for the C-terminal module. This suggests that *IRBP *arose through the quadruplication of an ancestral gene composed of 3 exons [25,27,28].

The African clawed frog, *Xenopus laevis*, offers unique advantages to studies of IRBP structure and function. The large size of its photoreceptors facilitates morphological analysis, and its eyecups are metabolically active for extended periods [29]. Unlike other amphibians, the *Xenopus *retina can be detached in both light- and dark-adapted animals. Remarkably, the detached retina may be re-constituted allowing the introduction of molecules into the adult subretinal space [30,31]. Molecules may also be introduced into the embryonic subretinal space through optic vesicle injections [32]. One of the most exciting advances is that the *Xenopus *retina is particularly amenable to transgenic manipulation [29,33-38]. Finally, X-ray crystallographic studies have recently determined the structure of the second module of *Xenopus *IRBP [39]. This advance taken together with the genetic and cellular advantages of *Xenopus *as an experimental system promise to uncover the mechanism of IRBPs function in the retina.

Here, we isolated and sequenced a full-length cDNA for *Xenopus IRBP*, and used this gene to express the individual modules, double modules, and the full-length *Xenopus *IRBP in *E. coli*. We generated atomic models of modules 1, 3 and 4 by homology modeling and docked all-*trans *retinol molecules in all four modules to examine the location and properties of possible ligand-binding sites. Our main finding is that although in terms of function and evolution the modules appear to represent fundamental units of IRBP, each module contains two qualitatively different ligand-binding domains. Furthermore, the corresponding domains between modules have different biochemical properties and non-identical local structures. Finally, interactions between the modules will likely provide important information toward understanding the structure of IRBP and its role in the visual cycle.

## Results

### Molecular cloning and sequence analysis

**Figure 1:**
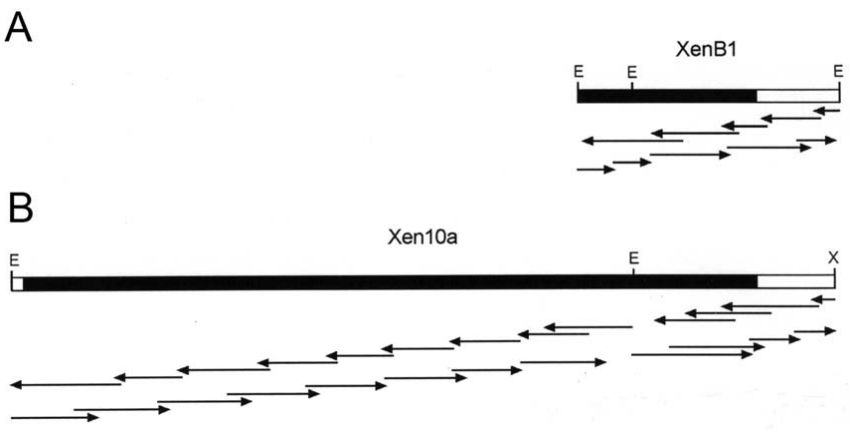
**Characterization of full-length cDNA for *Xenopus *IRBP**. **A) **cDNA XenB1 isolated under low stringency conditions [40], was used here to screen under high stringency a *Xenopus *stage 45 swimming tadpole cDNA library. **B) **This screen led to the isolation of Xen10a, a full-length *Xenopus *IRBP cDNA whose sequence is shown in Figure 2. Arrows represent individual sequencing runs. The solid bars indicate coding regions. E, EcoR1; X, XbaI.

Using a human IRBP cDNA as a probe, we previously isolated by low stringency hybridization screening a *Xenopus *IRBP cDNA (XenB1) corresponding to the fourth (C-terminal) module of the protein [40]. Here, XenB1 was used to screen under high stringency conditions a stage 45 *Xenopus *cDNA library enriched for full-length transcripts. The longest IRBP cDNA isolated (Xen10a) was 4,046 base pairs in length. This is consistent with Northern blot studies showing that the mRNA for *Xenopus *IRBPis approximately 4.2 kb in size [40]. The partial length XenB1, and full-length Xen10a cDNAs are shown diagrammatically in Figure 1 together with their sequencing strategies.

**Figure 2:**
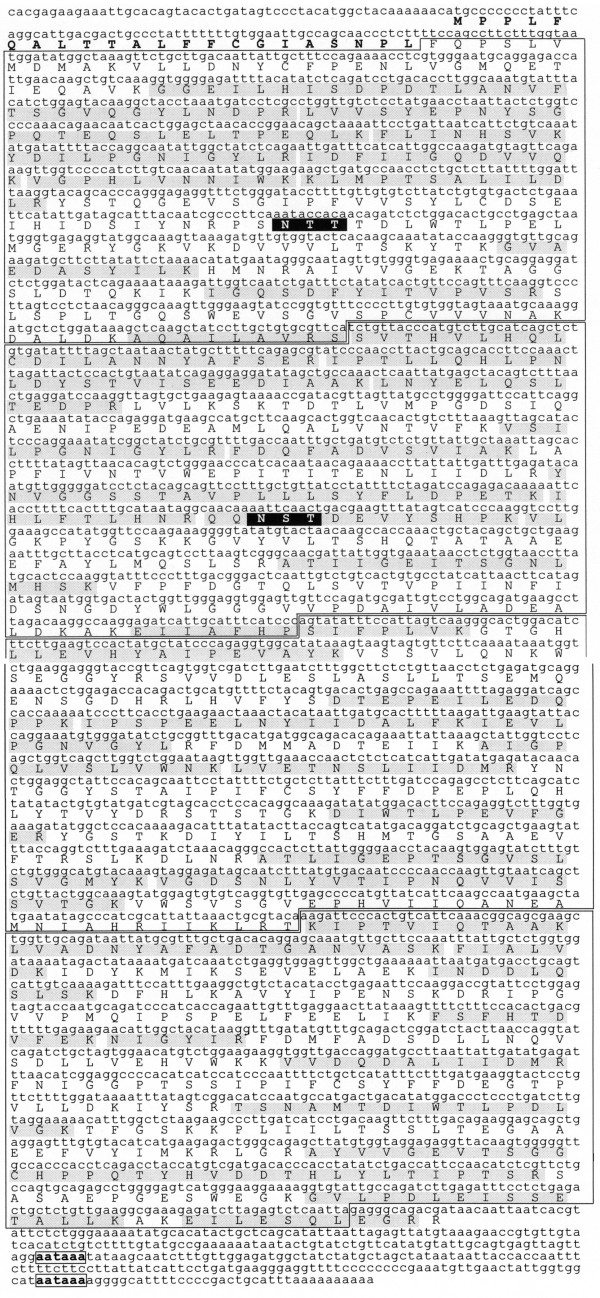
**Translated amino acid sequence of *Xenopus *IRBP**. The first 22 amino acid residues (bold) comprise the signal sequence. The four homologous modules of the protein are boxed. The black boxes with white text are glycosylation consensus sequences. The shaded regions are segments of the recombinant protein that were verified by LC-MS/MS. The 3'-UTR contains two polyadenylation signal sequences (boxed and in bold). This cDNA sequence is available through the European Bioinformatics Information, Genbank and DDBJ Nucleotide Sequence databases under accession number X95473 and sequence identification XLIRBP.

Xen10a codes for a polypeptide of 1197 amino acid residues (Figure 2). The entire protein without its signal peptide has a calculated molecular mass of 132,689Da. The sequence begins with a stretch of 22 N-terminal residues (bold in Figure 2) with features characteristics of signal recognition peptides [41]. The initiating methionine is followed by two amino acid residues, which have a net positive charge, and a hydrophobic domain ending in several polar residues.

**Figure 3:**
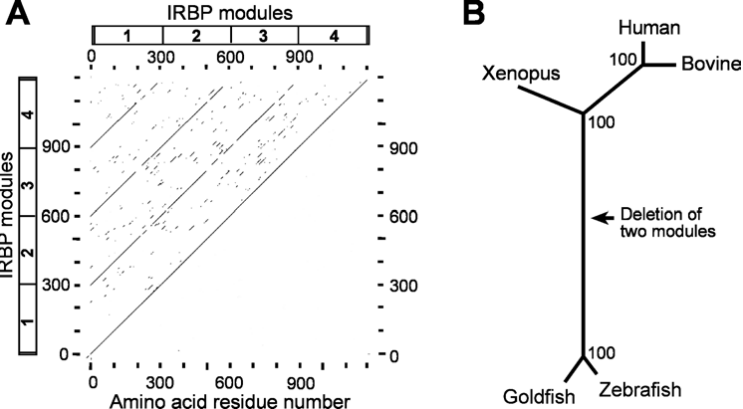
**Comparison of *Xenopus *IRBP with itself and known IRBPs**. **A) **Dotplot of *Xenopus *IRBP, comparing the protein sequence against itself. The boxes on the ordinate and abscissa are schematic diagrams of modules 1 through 4. The numbering on the axes correspond with the amino acid residues in Figure 2. The diagonal lines indicate regions of internal similarity, and hence the presence of the four modules. **B) **Distance tree showing the relationship between *Xenopus*, human, bovine, goldfish and zebrafish IRBPs. The branch lengths are drawn to scale and the values at the nodes indicate the number of times a grouping occurred in a set of 100 bootstrap values. The long distance separating the teleosts from amphibians is due in part to the teleosts having only two modules.

Following the signal peptide, the cDNA sequence consists of four homologous consecutive "repeats". Each repeat codes for a "module" of ~300 amino acid residues (boxed in Figure 2). All of the amino acid residues of *Xenopus *IRBP, excluding the signal peptide and the C-terminal four residues, can be assigned to one of the four homologous regions. This is illustrated in the dot-plot diagram of *Xenopus *IRBP compared to itself (Figure 3A). The overall structure of *Xenopus *IRBP is therefore similar to that of mammalian IRBPs which are also composed of four modules, and different from that of teleosts which contain only two modules. The relationship between the primary structure of human, bovine, *Xenopus*, goldfish and zebrafish IRBPs is shown by the distance tree in Figure 3B. The distance segment separating teleost and *Xenopus *IRBPs is considerably longer than that between *Xenopus *and mammalian IRBPs. The longer distance is due in part to the fact that teleost IRBP consists of only two modules. *Xenopus *IRBP is placed with a high level of confidence between that of mammals and teleosts in accordance with known phylogenetic relationships.

**Figure 4:**
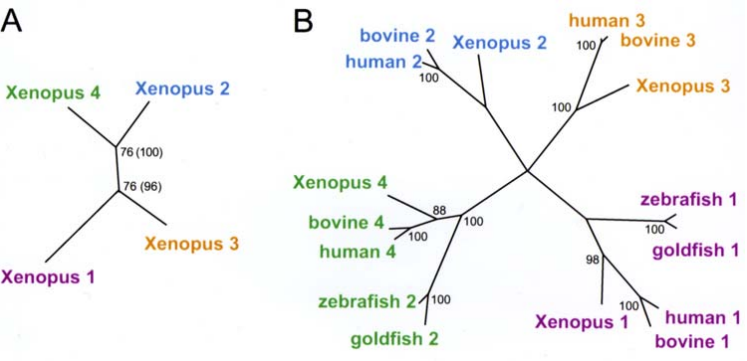
**Phylogenetic relationship of the IRBP modules**. **A) **Phylogenetic distances between various modules of *Xenopus *IRBP protein. The numbers at the junctions are the number of times a branch point occurred out of 100 bootstrap reiterations. The tree was rooted with module 4, the ancestral module. **B) **An unrooted distance tree showing the relationships between the various IRBP modules from different animals. The tree was constructed as in panel A. Even numbered modules are more closely related to each other than to odd numbered modules.

The phylogenetic relationship between each of the IRBP modules is shown in Figure 4. PanelA of this figure shows the phylogenetic distances between the various modules of *Xenopus *IRBP. The tree was rooted with module 4, the ancestral module [27,42]. The tree demonstrates that modules 2 and 4 are more closely related to each other than either is to modules 1 or 3. IRBP modules from different animals are compared by the unrooted distance tree in Figure 4B. The central branch point had a boot-strap value less than 80 and was collapsed into polytomy. A distance tree constructed with the Neighbor method gave the same general topography, except at the central branch point. A parsimony analysis also produced a tree with the same topography. Each of the *Xenopus *IRBP modules was most similar to the correspondingly numbered module of bovine and human IRBPs. In contrast, when compared to goldfish and zebrafish IRBPs, the fourth module of *Xenopus *and mammalian IRBPs were most similar to the second module of IRBP in teleosts (green labeling in Figure 4B). The N-terminal modules always showed the highest similarity between themselves in all of the species examined (purple labeling in Figure 4B). These observations are consistent with the suggestion that the middle two modules of teleost IRBP were lost during the evolution of the ray-finned fish (actinopterygii) [25].

Searches of the GenPept database with consecutive residue fragments of the Xenopus IRBP sequence showed a high level of similarity with other IRBPs in the database. In addition, each of the modules showed similarity with C-terminal processing proteases (CtpAs) [43], and enoyl-CoA hydratases (crotonases) [44,45] (data not illustrated). These observations taken together with X-ray crystallographic data from the second module of Xenopus IRBP suggest that IRBP belongs to the CptA/crotonase superfamily, and shares with other members of the family a common ligand-binding domain to stabilize unique hydrophobic molecules [1,39].

A glycosylation consensus site is present in modules 1 and 2 (white letters with black background in Figure 2). This observation is consistent with the fact that bovine IRBP contains asparagine-linked sialoligosaccharides [46]. Glycosylation differences between species could explain why Xenopus IRBP (M_r _= 124 kDa) [40] has a faster electrophoretic mobility on SDS-PAGE compared to that of bovine IRBP (M_r _= 145 kDa) [47,48]. The role of IRBP's sugar moieties, which appear unnecessary for its elongated shape, secretion, or ability to bind retinoids, is presently unknown [46].

The 3'-untranslated region of Xenopus IRBP, which is considerably shorter than that of mammalian IRBPs, contains two typical polyadenylation signal sequences (bold and boxed in Figure 2). The use of alternative signal sequences for in IRBP has been shown in the rat retina where two IRBP mRNAs of different lengths are expressed [49]. The significance of these observations to regulating IRBP mRNA stability is largely unknown.

### Full-length Xenopus IRBP: expression, retinol protection and binding parameters for all-trans retinol and 11-cis retinaldehyde

A remarkable characteristic of the IRBP-thioredoxin fusion proteins generated in this study was that they could be expressed to a significant extent in a soluble and biochemically active form in *E. coli*. The use of thioredoxin as a fusion partner was previously found to significantly enhance the soluble expression of X4IRBP [50] compared to X4IRBP without thioredoxin [51]. Here, we found that this effect could be extended to the other modules, and to the full-length Xenopus IRBP as well. A variety of proteins ranging from receptors to enzymes have been expressed in a soluble and active form as thioredoxin fusion proteins [52]. We found that optimizing the induction temperature can further enhance the expression of IRBP in the cytosol over that in the insoluble fraction. This effect, which is related to the reduced aggregation of recombinant proteins at lower expression temperatures, has been used to improve the solubility of a variety proteins in *E. coli* [reviewed in [53]]. Although the lower temperatures required extended incubation times, the final quantity of recombinant protein produced remained approximately the same as that generated at 37°C. The optimum incubation times and temperatures are given in Table 1. The recombinant proteins were purified by a combination of ammonium sulfate precipitation, ion exchange chromatography, and arsenical and Ni^2+^-affinity chromatography.

**Table 1:** *Summary of DNA oligonucleotides used to prepare the plasmid constructs, molecular masses, extinction coefficients, and yields of the purified IRBP-thioredoxin fusion proteins*. Except for module 4, which was cloned directly into the expression plasmid from a previously described cDNA (XenB1) (Gonzalez-Fernandez et al., 1993 J. Cell Sci. 105:7–21), cDNAs for IRBP and its individual modules were amplified by PCR from the full-length cDNA isolated in the present study. For each primer pair, the sense primer is written above the antisense primer. Regions of each primer corresponding to the plasmid multiple cloning segment are: acaaggtacccggggatcct (sense), and cttaaggtcgactctagagg (antisense). For each sense primer, the codon corresponding to the first amino acid residue of each module is underlined. For the antisense primer the stop codon is underlined.

Protein	Primer Sequence or cDNA	Vector (*E. coli*)	Induction Temp, time*	KDa†	ε (M^-1 ^cm^-1^)‡	Yield (per liter *E. coli*)
Full-length IRBP	ttccagccttctttggtaatttatcgtctgccctctaatt	pThioHis (Top10)	30°C, 5 hrs	148	131,420	7 mg (47 nmoles)
Module 1	ttccagccttctttggtaattgaacgcacagcaaggatag	pThioHis (Top10)	20°C, 21 hrs	47	48,010	15 mg (320 nmoles)
Module 2	tctgttacccatgtcttgcatgggatgaaatgcaatgatct	pThioHis (Top10)	30°C, 21 hrs	49	39,640	20 mg (410 nmoles)
Module 3	agtatatttccattagtcaagggctgtacgcagtttaataatgcg	pTrxFus (GI698)	30°C, 5 hrs	50	60,100	22 mg (460 nmoles)
Module 4	XenB1	pTrxFus (GI724)	35°C, 4 hrs	50	48,010	16 mg (320 nmoles)

* Calculated from the translated amino acid sequence including the thioredoxin fusion protein.

**† **The temperature and duration of protein induction was determined empirically from pilot studies.

‡ Molecular masses and protein extinction coefficients (ε) were calculated from the amino acid sequences and include the thioredoxin fusion protein (Gill and Von Hippel, 1989 Anal. Biochem. 182:319–326).

**Figure 5:**
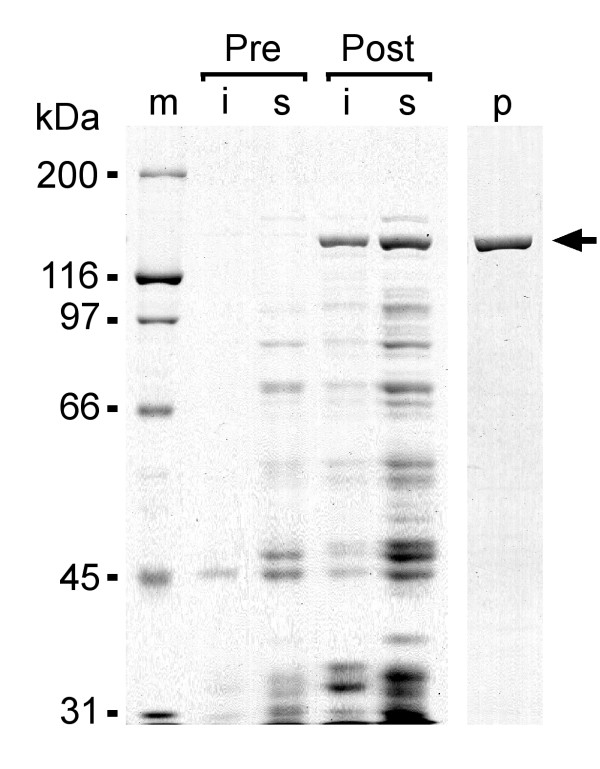
**Expression and purification of full-length *Xenopus *IRBP expressed in *E. coli *as a soluble thioredoxin/histidine-patch fusion protein (arrow)**. Coomassie blue stained 8% polyacrylamide gels, showing over expression of the recombinant IRBP. m = molecular weight markers; i = insoluble fraction; s = soluble fraction; "Pre" and "Post" refer to the bacterial fractions before and after induction with IPTG; p = purified protein.

Figure 5 illustrates the expression and purification of the full-length *Xenopus *IRBP-fusion protein. The overall yield of purified full-length *Xenopus *IRBP was 47 nmoles per liter of *E. coli *(Table 1). The sequence of the full-length *Xenopus *IRBP was confirmed by LC-electrospray-tandem mass spectrometry. For the full-length IRBP the mass/charge ratio was determined for 43 peptides released from an in gel trypsin digest of the fusion protein. The peptides had mass/charge ratios corresponding to predicted trypsin digest fragments. The locations of these fragments are indicated by gray shading in Figure 2. The sequences of 5 selected peptides were determined by LC-MS/MS. In all cases the amino acid sequences were identical to that predicted from the translated amino-acid sequence.

**Figure 6:**
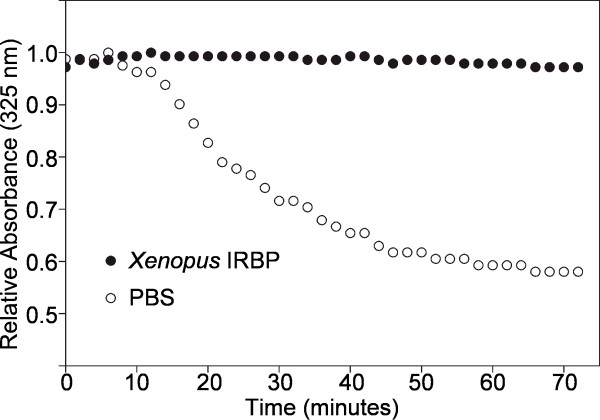
**Protection of all-*trans *retinol by full-length *Xenopus *IRBP**. Three μl of an ethanolic solution of all-*trans *retinol was added to 400 μl of 2.7 μM full-length *Xenopus *IRBP in PBS. The final concentration of all-*trans *retinol in solution was 3.2 μM. The degradation of all-*trans *retinol was monitored by measuring its absorbance at 325 nm as a function of time. For each sample, absorbance measurements were made every 2 min for 72 minutes. Full-length *Xenopus *IRBP (filled circles) is able to protect all-*trans *retinol from degradation as compared to a PBS control (unfilled circles).

The Xenopus-IRBP fusion protein, which is to our knowledge represents the first full-length IRBP generated in a prokaryotic system, binds and stabilizes visual-cycle retinoids. In Figure 6 the absorbance of the protein with bound all-trans retinol is monitored at 325 nm (λ_max _of retinol) as a function of time. Over 72 min., only a small drop in absorbance was appreciated. This ability to maintain the absorbance at 325 nm is characteristic of bovine IRBP, and may be related to an activity in maintaining the oxidation state of retinol [11].

The binding parameters of retinoid-binding proteins including IRBP may be determined by fluorescence spectroscopy. The method utilizes changes in quantum yield occurring during ligand binding [54]. Such changes are represented by enhancement of ligand fluorescence, quenching of intrinsic protein fluorescence, and transfer of energy from protein to bound ligand. Fluorescence enhancement occurs if the quantum yield of the ligand's fluorescence is higher in the hydrophobic environment of the ligand-binding domain compared to that outside of the domain.

**Figure 7:**
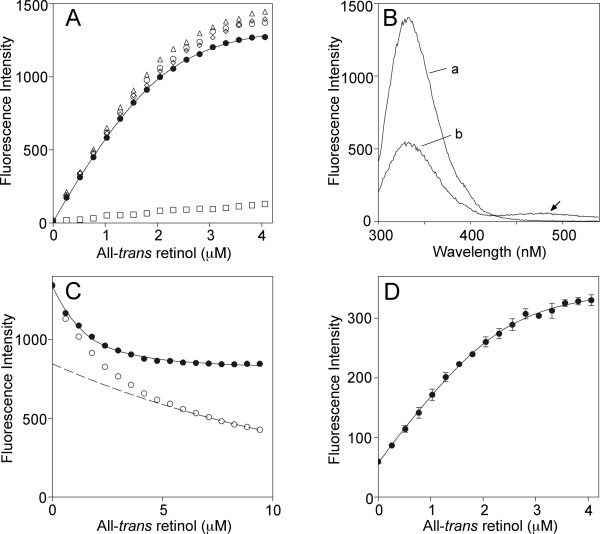
**Fluorescence-binding studies of full-length *Xenopus *IRBP to all-*trans *retinol**. The concentration of *Xenopus *IRBP was 0.65 μM in each panel. **A) **Titrations of IRBP with all-*trans *retinol as followed by monitoring the increase in retinol fluorescence (excitation, 330 nm; emission, 480 nm). Retinol fluorescence in the presence of IRBP (○,◊, Δ) is compared with that in the presence of an fluorescence matched solution of N-acetyl-L-tryptophanamide (□). The difference between these two curves, the fluorescence enhancement (-●-), represents all-*trans *retinol bound to the protein. The curve is a nonlinear least squares fit of Equation 1 to the binding data. Error bars are too small to be visualized. The number of binding sites per molecule of protein (*N*) was 3.19 ± 0.10 with *K*_*d*_^*all-trans *^= 0.30 ± 0.05 μM (standard error of the mean). **B)**. Emission spectra of apo- and holo-IRBP (curves a and b, respectively) were obtained upon excitation at 280 nm in the presence of a 10 fold excess of all-*trans *retinol. The drop in emission at 340 nm represents quenching of the protein's intrinsic fluorescence. The emission at 480 (arrow) represents energy transfer to the bound retinol. **C) **Titration monitoring quenching of intrinsic protein fluorescence by bound retinol. Excitation and emission wavelengths were 280 and 340 nm, respectively. The inner filter effect has been accounted for graphically as previously described (50) (-○-, actual meausurements; -●-, after correction). Calculated binding parameters: *N *= 1.93 ± 0.41; *K*_*d*_^*all*-*trans *^= 0.66 ± 0.14 μM. **D) **Titration monitoring energy transfer (increase in fluorescence at 480 (arrow). Calculated binding parameters: N = 3.72 ± 0.20; *K*_*d*_^*all*-*trans *^= 0.29 ± 0.12.

Figure 7 shows fluorescence titrations and spectra of all-*trans *retinol binding to full-length *Xenopus *IRBP. In panel A, binding was followed by monitoring retinol fluorescence enhancement. The enhancement was determined by subtracting the fluorescence of retinol in the presence of an OD_280 _matched solution of N-acetyl-L-tryptophanamide from that in the presence of IRBP. N-acetyl-L-tryptophanamide, which from its indole ring has a typical protein-like fluorescence, serves as a blank because it does not significantly interact with retinol [55]. Equation 1 was fit to the data by nonlinear least squares analysis. The number of binding sites per molecule of IRBP was calculated to be 3.19 ± 0.10 with *K*_*d *_= 0.30 ± 0.05 μM (Table 1I). Figure 7B shows emission spectra of apo- and holo-*Xenopus *IRBP (excitation = 280 nm). The lower emission of the holoprotein at 340 nm represents protein quenching mainly due to tryptophan. Panel C shows the titration monitoring the decrease in fluorescence at 340 nm (open circles). A nonspecific decrease in fluorescence at this wavelength, which is largely due to the inner filter effect, is accounted for by graphical correction (dashed line) as previously described [56]. Fitting equation 1 to the data gives *N *= 1.93 ± 0.41, and *K*_*d *_= 0.66 ± 0.14 μM. Finally, Figure 7D monitors energy transfer (excitation, 280; emission, 480 nm) giving *N *= 3.72 ± 0.20, and *K*_*d *_= 0.29 ± 0.12 μM.

**Figure 8:**
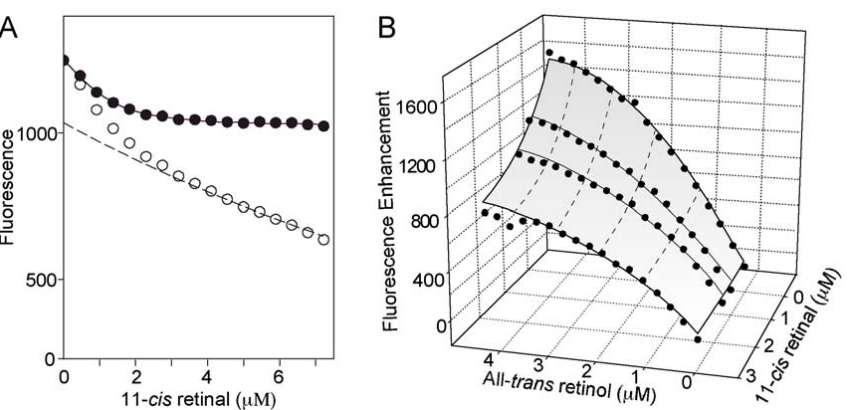
**11-*cis *retinaldehyde binding to full-length *Xenopus *IRBP**. Since retinaldehydes are nonfluorescent compounds their binding cannot be followed by ligand fluorescence enhancement or energy transfer. Here, the binding of 11-*cis *retinaldehyde is followed by monitoring quenching of endogenous protein fluorescence, and indirectly by competition with the efficient fluorophore all-*trans *retinol. **A) **Representative titrations monitoring quenching of intrinsic protein fluorescence by bound 11-*cis *retinaldehyde. Excitation and emission wavelengths were 280 and 340 nm, respectively. The inner filter effect was accounted for by graphical correction as previously described [56] (-●-, before correction for inner filter effect; -●-, after correction). The binding parameters were calculated to be: *N *= 1.81 ± 0.15; *K*_*d*_^11-*cis *^= 0.28 ± 0.05 μM. **B**. Representative competition titration. From each titration the emission of an 0.D._280 _matched solution of N-acetyl-L-tryptophanamide was subtracted. Binding parameters: *N *= 3.53 ± 0.19 with *K*_*d*_^*all*-*trans *^= 0.22 ± 0.13 μM; *K*_*d*_^11-*cis *^= 0.21 ± 0.10 μM.

We were interested in asking whether *Xenopus *IRBP or its individual modules (see below) can discriminate between all-*trans *retinol and 11-*cis *retinaldehyde. Compared to studies using retinol, monitoring 11-*cis *retinaldehyde binding is more challenging as it is not significantly fluorescent precluding measurements of fluorescence enhancement. However, its binding can be measured by following protein quenching, and its ability to displace all-*trans *retinol. Figure 8A shows a titration of 11-*cis *retinaldehyde to the full-length *Xenopus *IRBP monitoring protein quenching. The number of binding sties were calculated to be 1.81 ± 0.15 with a *K*_*d *_^11-*cis *^= 0.28 ± 0.05 μM (Table 3).

**Table 3:** *Summary of 11-cis retinal binding parameters to IRBP*. The binding of 11-*cis *retinal to full-length IRBP and the four modules was determined by competitive fluorescence spectroscopy using all-*trans *retinol.

Protein	Binding sites (N)	K_d _(μM) All-*trans *retinol	K_d _(μM) 11-*cis *retinal
Full-length	3.53 ± 0.19	0.22 ± 0.13	0.21 ± 0.10
Module 1	1.29 ± 0.24	0.67 ± 0.17	0.57 ± 0.11
Module 2	1.22 ± 0.10	0.074 ± 0.037	0.28 ± 0.13
Module 3	1.90 ± 0.28	0.84 ± 0.22	0.56 ± 0.11
Module 4	1.47 ± 0.18	0.41 ± 0.09	0.29 ± 0.05

In Figure 8B, full-length *Xenopus *IRBP was titrated with all-*trans *retinol in the presence of different fixed concentrations of 11-*cis *retinaldehyde. Binding of all-*trans *retinol to *Xenopus *IRBP was followed by monitoring the enhancement of its fluorescence. Note that increasing the concentration 11-*cis *retinaldehyde resulted in a reduction in the level of all-*trans *retinol fluorescence enhancement (Figure 8B). Equation 3 (see methods) was used to analyze the data generating the 3-dimensional representation of the fit shown graphically in Figure 8B. The number of binding sites was calculated to be 3.53 ± 0.19 with *K*_*d *_^*all*-*trans *^= 0.22 ± 0.13 μM and *K*_*d *_^11-*cis *^= 0.21 ± 0.10 μM (Table 3).

### Expression and ligand-binding properties of the individual Xenopus-IRBP modules

Saturable binding for one to two all-*trans *retinol equivalents could be detected for each module. Often fewer sites were detected by titrations following ligand quenching of protein fluorescence compared to that monitoring retinol-fluorescence enhancement. This is because quenching requires the presence of a tryptophan in the ligand-binding domain. In contrast, this residue is not required to support ligand-fluorescence enhancement. The binding parameters for all-*trans *retinol derived from enhancement and quenching titrations are shown in Table 2 for each module, and the full-length *Xenopus *IRBP. The equilibrium dissociation constants ranged from 0.084 ± 0.022 μM (module 2) to 1.79 ± 0.54 μM (module 4) (enhancement, Table 2). Monitoring fluorescence enhancement, two sites could be detected in modules 3 and 4 (1.83 ± 0.42, 2.06 ± 0.53 sites respectively). 1.57 ± 0.04 were detected in module 2. In contrast, only one site (*N *= 0.83 ± 0.15) could be detected in module 1. Fewer sites were detected in titrations monitoring quenching. For modules 1, 3 and 4, less than one site was detected. Only for module II was a full site detected by quenching (*N *= 1.31 ± 0.06). Thus, as with the full-length *Xenopus *IRBP, fewer of the ligand-binding domains could be detected by fluorescence quenching compared to assays monitoring retinol fluorescence enhancement.

**Table 2:** *Summary of all-trans retinol binding parameters to IRBP*. The binding of all-*trans *retinol to full-length IRBP and the four modules was determined by measuring the enhancement of all-*trans *retinol fluorescence and the quenching of protein endogenous fluorescence as a function of retinol concentration.

Protein	Binding sites (N) K_d _(μM)	Enhancement^*a*^	Quenching^*b*^
Full-length IRBP	N =	3.19 ± 0.10	1.93 ± 0.41
	Kd =	0.30 ± 0.05	0.66 ± 0.14
Module 1	N =	0.83 ± 0.15	0.25 ± 0.10
	Kd =	0.44 ± 0.13	0.35 ± 0.08
Module 2^*c*^	N =	1.57 ± 0.04	1.31 ± 0.06
	Kd =	0.084 ± 0.022	0.14 ± 0.04
Module 3	N =	1.83 ± 0.42	0.65 ± 0.08
	Kd =	1.18 ± 0.41	0.31 ± 0.04
Module 4	N =	2.06 ± 0.53	0.12 ± 0.05
	Kd =	1.79 ± 0.54	0.64 ± 0.05

^*a *^Monitored at 480 nm upon excitation at 330 nm.

^*b *^Monitored at 340 nm upon excitation at 280 nm.

^*c *^Module 2 data from Loew *et al*. *Exp Eye Res*. 2001, **73**:257–264

**Figure 9:**
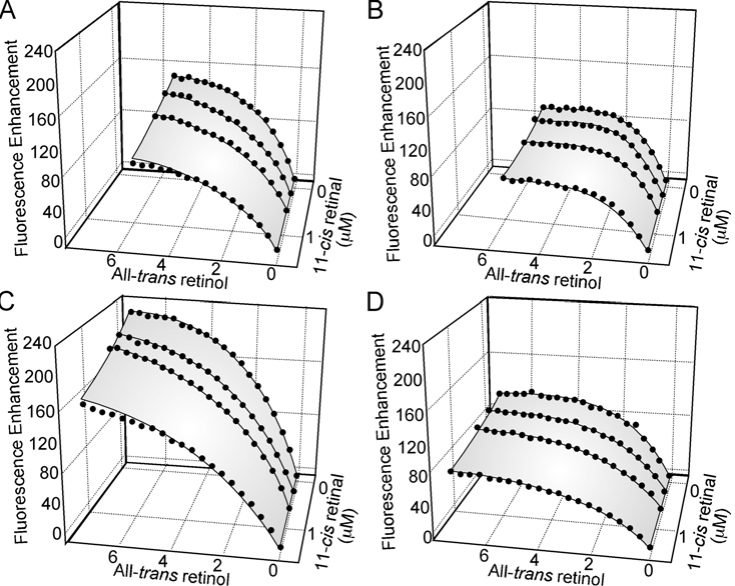
**Competitive inhibition by 11-*cis *retinaldehyde of all-*trans *retinol binding to the individual modules of *Xenopus *IRBP**. Binding of all-*trans *retinol to the individual modules was measured by fluorescence enhancement in the presence of varying amounts of 11-*cis *retinal. Panels A through D correspond to modules 1 through 4 respectively. Assuming both all-*trans *retinol and 11-*cis *retinal share the same binding sites, all-*trans *retinol binding to IRBP is competitively inhibited. Three dimensional nonlinear regression was used to determine the number of binding sites, the dissociation constant of all-*trans *retinol, and the dissociation constant of 11-*cis *retinal for each of the individual modules. The binding parameters are summarized in Table 3. The concentrations of the proteins used in the titrations were as follows: module 1 (0.99 μM; panel A); module 2 (1.10 μM; panel B); module 3 (1.10 μM; panel C); module 4 (1.00 μM; panel D).

The binding of 11-*cis *retinaldehyde was characterized by competition with all-*trans *retinol (Figure 9, Table 3). The binding parameters for all-*trans *retinol were similar with those in Table 2 except for module 4 where the K*d *was 0.41 ± 0.09 μM compared to 1.79 ± 0.54 μM in the absence of 11-cis retinaldehyde. The reason for this difference is not clear, but may represent an allosteric interaction in this module.

For the individual modules and full-length *Xenopus *IRBP, the equilibrium dissociation constants for all-*trans *retinol, and 11-*cis *retinaldehyde were similar except for module 2. Here, the K*d *for all-*trans *retinol and 11-*cis *retinaldehyde were significantly different (0.074 ± 0.037 μM and 0.28 ± 0.13 μM respectively). This suggests that module 2 has selectivity for all-*trans *retinol over 11-*cis *retinaldehyde. Molecular modeling studies described below, indicate that the putative sites may provide highly ordered environments perhaps discriminating between the isomeric configuration, oxidative state, or both of the ligand. Ongoing studies will address these possibilities by preparing crystals of X2IRBP with bound ligand.

**Figure 10:**
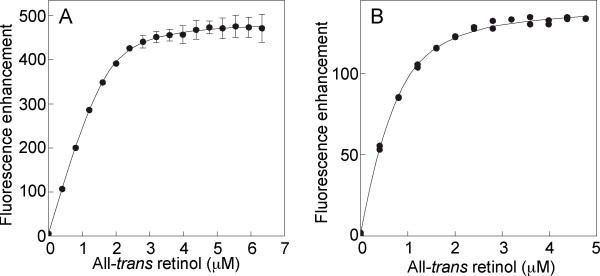
**Retinol binding to *Xenopus *IRBPs consisting of two contiguous modules**. Binding of double modules IRBPs with all-*trans *retinol as followed during titrations by monitoring the increase in retinol fluorescence (excitation, 330 nm; emission, 480 nm). The concentration of the IRBP double module was: modules 1&2, 0.67 μM; modules 3&4, 0.57 μM. **A) ***Xen *IRBP modules 1&2 showed *N *= 2.45 ± 0.11 with *K*_*d *_= 0.049 ± 0.023 μM. **B) ***Xen *IRBP modules 3&4 showed N = 1.43 ± 0.21 with *K*_*d *_= 0.19 ± 0.05 μM.

Why do the number of sites from individual modules add up to more than that detected in the full-length IRBP? Here we asked whether the number of sites within individual modules could at least predict the number in recombinant proteins consisting of two modules. We had originally anticipated that the sum of sites from individual modules would give the number in such "module pairs". However, this turned out to be not always true. We expressed and purified module combinations 1&2 and 3&4, termed X(1,2)IRBP and X(3,4)IRBP respectively. Binding of all-*trans *retinol to these module pairs was characterized by fluorometric titrations monitoring retinol-fluorescence enhancement (Figure 10). For X(1,2)IRBP, *N *= 2.45 ± 0.11 (2.40 sites were predicted from the sum of individual modules 1 plus 2). In contrast, *N *for X(3,4)IRBP was 1.43 ± 0.21 sites (predicted, 3.83 sites). These results are consistent with the notion that some of the sites within individual modules are cryptic in the intact full-length IRBP. That is, some sites may not be solvent exposed in the intact IRBP as they are within individual modules. Interestingly, the affinity for retinol was greater in the module pair compared to that in the individual module. The *K*_*d*_'s for modules 1 and 2 were 0.44 and 0.084 respectively. In contrast, the *K*_*d *_for X(1,2)IRBP was 0.049 ± 0.023 μM. Similarly, the *K*_*d *_'s for modules 3 and 4 were 1.18 and 1.79 μM respectively. In contrast, the *K*_*d *_for X(3,4)IRBP was 0.19 ± 0.05 μM. These observations suggest that the presence of more than one module may be required for the specificity of individual ligand-binding domains. Since the existing X-ray crystal structure of IRBP is limited to only a single module (X2IRBP), we do not know the quaternary structure, or how the modules fit together to form the intact full-length IRBP. Therefore it is plausible that some binding sites that are solvent exposed in the individual module are buried in the intact module. To address this question, ongoing studies in our laboratories are aimed at determining the X-ray crystal structure of IRBPs composed of multiple modules.

To further characterize the recombinant *Xenopus *IRBP and it modules, the fluorescent stearic acid analog, 9-(9-anthroyloxy) stearic acid (9-AS) was used as a probe (Table 4). 9-AS has been previously used to study the fatty-acid binding properties of native bovine IRBP [58]. Monitoring the enhancement of 9-AS fluorescence upon binding to the full-length *Xenopus *IRBP showed 2.58 ± 0.17 sites with *K*_*d *_= 0.28 ± 0.07 μM. Although similar to the affinity for all-*trans *retinol (0.30 ± 0.05 μM), the number of sites detected with retinol was somewhat greater (3.19 ± 0.10 sites) (Table 2). Interestingly, the number of sites detected by monitoring protein quenching with all-*trans *retinol (1.93 ± 0.41 sites) was also greater than that detected by quenching with 9-AS (0.18 ± 0.29 sites). Surprisingly, most of the individual modules had a similar binding capacity for 9-AS compared to that of the full-length protein (Table 4). This suggests the possibility that the fewer sites detected with 9-AS compared to that with all-*trans *retinol in the full-length *Xenopus *IRBP is due to the loss of a site typically supporting both fluorescence enhancement and quenching in the individual module. Such a site appears to be present in the individual modules, but can no longer be probed with 9-AS in the full-length *Xenopus *IRBP. Ongoing structural studies of the full-length IRBP should help to define the relationship of these ligand-binding sites in the full-length IRBP.

**Table 4:** *Summary of 9-AS binding parameters to Xenopus IRBP as determined by fluorescence spectroscopy*. The binding of 9-AS to full-length IRBP and the four modules was determined by measuring the enhancement of 9-AS fluorescence and the quenching of protein endogenous fluorescence as a function of 9-AS concentration.

Protein	Binding sites (N) K_d _(μM)	Enhancement^*a*^	Quenching^*b*^
Full-length IRBP	N =	2.58 ± 0.17	0.18 ± 0.29
	K_d _=	0.28 ± 0.07	0.29 ± 0.11
Module 1	N =	2.55 ± 0.24	0.97 ± 0.19
	K_d _=	0.33 ± 0.15	0.42 ± 0.10
Module 2^*c*^	N =	1.49 ± 0.15	0.93 ± 0.06
	K_d _=	0.25 ± 0.08	0.13 ± 0.02
Module 3	N =	2.76 ± 0.09	1.06 ± 0.07
	K_d _=	0.16 ± 0.41	0.19 ± 0.03
Module 4	N =	2.23 ± 0.09	0.30 ± 0.09
	K_d _=	0.082 ± 0.037	0.16 ± 0.04

^*a *^Monitored at 440 nm upon excitation at 360 nm.

^*b *^Monitored at 340 nm upon excitation at 280 nm.

^*c *^Module 2 data from Loew *et al*. *Exp Eye Res*. 2001, **73**:257–264.

### Ligand docking and homology modeling

**Figure 11:**
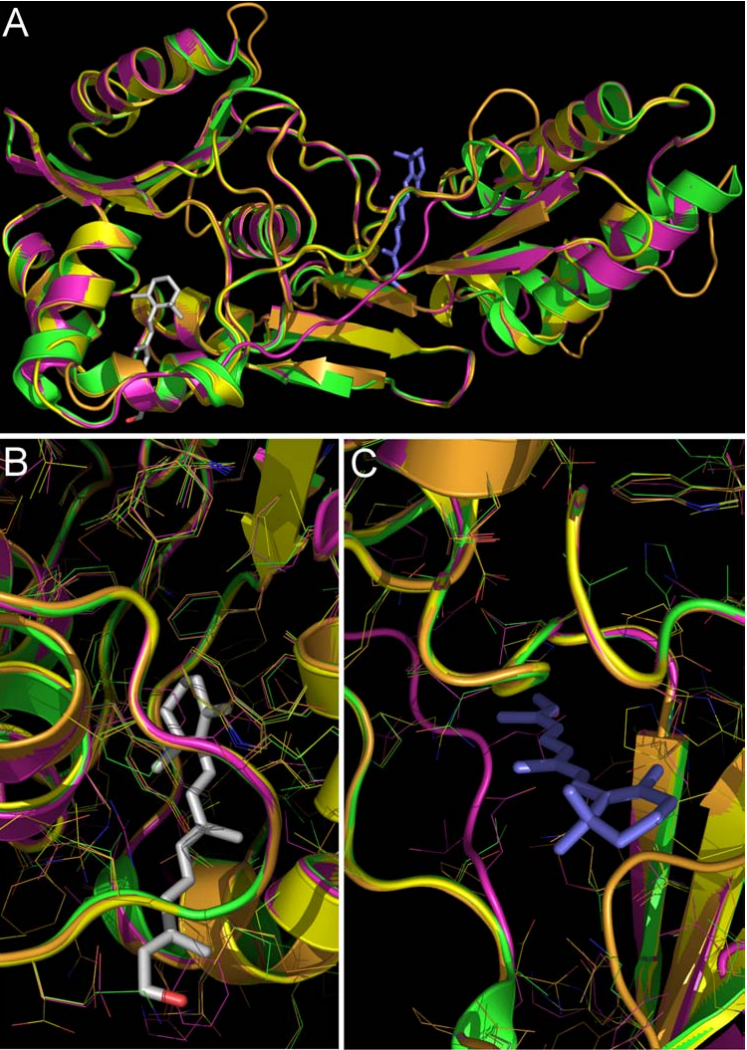
**Homology modeling of the modules of *Xenopus *IRBP**. **A) **Superimposition of the predicted structures of X1-, X3- and X4IRBPs with the X-ray crystal structure of X2IRBP. Ribbon diagram: X1IRBP, orange; X2IRBP, green; X3IRBP, magenta; X4IRBP, yellow. Molecular docking studies predict two binding sites for all-*trans *retinol. The best scoring poses for the conformational search are shown with the retinol molecule colored grey in site I, and blue in site II. Close-up views of the docked all-*trans *retinol molecule in (**B**) site I, and (**C**) site II. All side chains are shown by thin lines in the colors of the backbones. The amino acid residues within contact distances to the ligand are tabulated in Table 5.

As structural data are not available for any of the modules besides that of X2IRBP, we used homology modeling to compare the structures of the individual modules. First, molecular docking was used to predict the location of the all-*trans *retinol binding sties in the known X2IRBP structure. The *site finder *routine identified the largest two cavities to have sizes 79 and 74 (in arbitrary units, designated as sites I and II, respectively; the next cavity had a size of 42). Automated docking procedure and conformational analysis yielded two best scoring poses for all-*trans *retinol, one at each site. These poses are shown in Figure 11. In this figure, a docked molecule of all-*trans *retinol is colored grey in site I, and blue in site II. Site I, which is described by residues Ile80, Val99, Phe100, Phe114, Gln116, Phe117, Ala118, Ile123, Leu126, Ala127, Ile130, Val131, Trp135, Ala158, Leu161, Leu162, Tyr165 and Leu196, is highly hydrophobic and consists of residues residing almost entirely within the N-terminal A domain [51]. However, site II, delineated by residues Pro62, Arg63, Val65, Lys67, Asp71, Thr72, Leu73, Ser239, Gly240, Met243, His244, Ser245, Val247, Thr258, Leu273, Gly274 and Gly275, has a number of polar side chains and is situated at the interface between the A and the C-terminal B domains [51]. Site II, the hydrophobic region between the two domains was previously thought to represent the putative ligand-binding site within X2IRBP [39]. Both sites I and II have a nearby Trp residue, conserved among the functional units. It is therefore possible that both sites could support protein quenching.

For modeling structures of X1-, X3- and X4IRBP modules by homology, the X2IRBP crystal structure was selected by MOE as the best candidate through its automated PDB database search and sequence alignment routines. Figure 11a shows the overall superimposed modeled structures of modules X1-, X3-, and X4IRBP with the experimental X2IRBP structure. Overall, the superposition demonstrates high homology among three-dimensional structures of all four modules, particularly among the α helices and β sheets. Furthermore, from the superimposed structures it is clear that each module retains the locations of both of the two cavities described above as the putative ligand-binding sites predicted from the ligand-docking analysis (Figure 11b,c).

The amino acid residues that line each of these cavities are summarized in Table 5. This table lists the residues that are conserved in the putative ligand-binding pockets between each module, and those that are chemically different and/or would be predicted to sterically hinder retinol entry compared to the corresponding amino acid residue in X2IRBP. Upon comparing the domains, it is apparent that not only are sites I and II distinct, but the corresponding sites in four modules also appear to display some differences. First, site I has a significantly more hydrophobic character compared to site II. Secondly, site II, which has a higher percentage of polar residues, shows a more open binding pocket with less conservation of the residues contributing to the ligand-binding domain compared to site I.

**Table 5:** Comparison of the Site I and II ligand-binding domains within each IRBP module

Amino acid residues lining the two domains:														
	***Site I***							***Site II***						
		
X1:	W132	Y162	V97	I111	L127	I131	L109	W268	P63	S271	G236	Y73	T240	L238
X2:	W135	Y165	F100	F114	I130	V134	L112	W272	P62	G275	N241	L73	S245	M243
X3:	W133	Y163	F98	F112	L128	V132	L110	W271	H63	S274	V239	E75	V243	L241
X4:	W127	Y157	F92	F106	L122	V126	I104	W265	F55	L268	L233	S66	P237	H235
*Amino acid residues that are significantly different and/or interfering with ligand-binding*:														
	***Site I***							***Site II***						
				
X1:	V159	M191	E189	E121	L187			Y73	P76					
X2:	L162	L196	K194	A124	H192			L73	P76					
X3:	F160	F194	E192	K122	L190			E75	L77					
X4:	F154	V188	D186	N116	L184			S66	R69					

Structural differences between the sites in different modules are apparent upon comparing the amino acid residues contributing to these two putative ligand-binding sites. In particular, at several positions, residues within site I of X2IRBP have been replaced by bulkier residues. Leu162 has been replaced by phenylalanines in modules 3 and 4 (Phe160 and Phe154 respectively), which cause steric clash with the modeled retinol molecules. It is, thus, likely that in modules X3IRBP and X4IRBP, site I for retinol binding does not exist. A difference at this position is also noted for module 1 where the corresponding Leu162 has been replaced by Val159.

Site II, which is formed as a cleft between domains A and B on antiparallel β-sheets between residues 245 and 260, and the loop between residues and 241 and 245, also shows differences among modules. Residues of other modules that may interfere with the entry of retinol into site II are X1IRBP, Leu65; X3IRBP, Glu75, Leu77; X4IRBP, Phe55, Arg69. Another notable difference is that the loop in module 1 between Pro63 and Pro71 has a conformation different from those in modules X2, X3 and X4. In X1IRBP the loop has moved into the site II ligand-binding pocket. Finally, the loop between amino acid residues 75 to 83 in X3IRBP is dissimilar compared to the corresponding loops in the other three modules.

## Discussion

IRBP's structure consisting of internal homologous modules is unprecedented for a hydrophobic ligand-binding protein [59,60]. Extra- and intracellular retinoid-binding proteins typically do not have significant internal homology with duplication of ligand-binding domains, and bind only one ligand equivalent per polypeptide [61,62]. This is also generally true of the fatty acid-binding proteins, and other members of the calycin superfamily [63]. It is possible that IRBP's interesting module structure is related to the profound complexity of vitamin A trafficking in vision compared to other biological systems requiring extracellular retinoid transport. For example, serum retinol-binding protein transports only one chemical type of retinoid, all-trans retinol, in a unidirectional manner from the liver to the RPE. In contrast, IRBP is thought to mediate the bidirectional transport of several retinoid types (all-trans retinol, 11 cis retinal, and 11-cis retinol) between four cell types (rods, cones, RPE and Müller cells). It is therefore plausible that IRBP's module structure may have a role in targeting the correct retinoid to the intended cell type while protecting it from isomeric and oxidative degradation. Our long-term goal is to uncover the relationship between the structure and function of IRBP in the visual cycle. Here, we focused on the Xenopus IRBP homolog because an X-ray crystal structure is available for the second module in this species [39]. Furthermore, we anticipate that Xenopus will provide a valuable system to study the function and trafficking of IRBP in the retina using transgenic approaches.

The translated amino acid sequence of Xenopus IRBP begins with a signal peptide suggesting that the synthesis of the nascent IRBP begins with its insertion into the endoplasmic reticulum. Furthermore, N-terminal sequencing of IRBP from several species indicates that the signal peptide is cleaved at a limited number of sites. Although N-terminal sequence data of native Xenopus IRBP is not available, frog IRBP is known to have a 3 amino acid residue extension [64]. The same cleavage position would be predicted for the Xenopus IRBP signal peptide [65]. The above observations are consistent with immunohistochemical and biochemical studies showing that Xenopus IRBP accumulates in the IPM following its synthesis by the rods and cones [9,31,40,66,67].

Western blot studies have shown that native Xenopus IRBP (M_r _= 124 kDa) [40] is closer in size to IRBPs in mammals (M_r _= 146–135 kDa) [24,68-70] than to that in teleost fish (M_r _= 65–75) [25]. These observations are explained by the present finding that Xenopus IRBP is composed of 4 homologous segments each consisting of ~300 amino acid residues.

The Xenopus IRBP modules are related in specific ways to each other and to known IRBPs. Comparing the Xenopus modules to themselves, 2 & 4, and 1 & 3 are more similar to each other. A model to explain this relationship begins with the proposal that the IRBP gene arose through the insertion of a reverse transcribed processed mRNA 5' to the original gene [27]. After this first duplication event, the amino acid sequences of the two resulting modules diverged. A second duplication event occurring in the same way as the first could explain why similar and dissimilar modules alternate with one another [1]. That is, a reverse transcribed processed mRNA was inserted 5' to the two repeat IRBP. This second duplication probably occurred before, or early during the emergence of vertebrates since the size of skate and ray IRBPs suggests that IRBP in elasmobranchs is composed of 4 modules [71,72]. The quadruplication therefore occurred before the emergence of bony fish (Osteichthyes) since cartilaginous fish (Chondrichthyes), which include elasmobranchs, diverged before the emergence of the bony fish [73]. The dimodular teleost IRBP would then represent the loss of two repeats during the emergence of the ray finned fish (Actinopterygii). The similarity of the N- and C-terminal modules of teleost IRBP with that of Xenopus and mammals suggests that it was the middle two repeats that were lost. The deletion probably occurred by homologous recombination, a common mechanism for the elimination of DNA segments during the evolution of complex proteins [74,75]. This model could be tested by examining IRBPs of extant early Actinopterygii, the Sarcopterygii fish (the direct ancestors of amphibians), and the jawless fish (Agnatha).

The above model suggests that the IRBP modules contain an important function that was copied during the evolution of its gene. Similar yet non-identical repeats, suggests that the duplication events allowed for functional divergence. We therefore anticipated that each module should have functional activity, yet this activity would not be identical between modules. To study the modules independently, we expressed them separately, as pairs, and as the full-length protein.

Full-length recombinant *Xenopus *IRBP had the expected biochemical properties although the number of binding sites was somewhat higher than that of native bovine IRBP. Previous studies of bovine IRBP have reported values for *N *ranging from 1 to 2.6 sites per polypeptide. A more recent study found evidence for 3 ligand-binding sites in bovine IRBP [76]. This is consistent with the present study where we detected 3.19 ± 0.10 sites in *Xenopus *IRBP.

Surface hydrophobicity representations of X2IRBP call attention to two hydrophobic regions representing candidate ligand-binding domains [39]. Evidence that at least one of these domains (site II) may have biological significance comes from its structural homology with the active site of crotonases [77]. Superimposition of the structure of X2IRBP with that of 2-enoyl-CoA hydratase complexed with octanoyl-CoA suggests that site II in IRBP corresponds to the crotonase ligand-binding domain [39]. Although the precise biological function of site II in IRBP is not established, molecular docking analysis placed a molecule of all-*trans *retinol into its large shallow cleft. Interestingly, the docking analysis also positioned an all-*trans *retinol molecule within the more deeply buried N-terminal hydrophobic domain of site I. These observations are consistent with studies showing that in bovine IRBP, retinol is stabilized mainly by hydrophobic interactions [78]. Nevertheless, there is evidence from the structure of site I that retinol's OH group could form a hydrogen bond to Lys194 located nearby on the surface of X2IRBP. Finally, our data are consistent with studies of human IRBP showing that each module has one to two ligand-binding sites [79-81].

Our homology modeling studies suggest that the two putative ligand-binding sites identified by X-ray crystallography of X2IRBP are conserved in each of the other three modules of IRBP. Although the significance of the specific details awaits further experimental data, we can say that the two sites based on their overall morphology and local environment are structurally distinct and may therefore have significantly different functions. It is highly likely that not all four modules are involved in the same functional role of ligand-binding and/or transportation with similar efficiency. The amount of fluorescence enhancement is different for the various modules. The modules are also not equivalent in terms of their dissociation constants. The range of *K*_*d *_^*all*-*trans *^s could reflect unique demands of the visual cycle for supporting different levels of extracellular all-*trans *retinol flux occurring during scotopic and photopic conditions. Of particular interest is the apparent specificity of module 2 for all-*trans *retinol over 11-*cis *retinaldehyde. Taken together, our results suggest that the entire polypeptide chain possibly functions as a single protein with multiple modules performing various tasks of binding, protection and transportation of ligands, or yet another unknown role. Finally, one or both binding-sites may border other modules in the full-length IRBP. Thus, physiologically triggered changes in the quaternary structure of IRBP may allow significant changes in the availability of specific ligand-binding domains.

## Conclusion

IRBP has a remarkable structure consisting of repeats or modules of which there are four in *Xenopus*. This structure is probably critical to understanding the role of IRBP in the complicated physiology of retinoid trafficking between rods, cones, RPE, and Müller cells in the visual cycle. Our studies suggest that the module structure not only provides for increased ligand carrying capacity, but also allows for qualitative differences in the affinity, and specificity of the binding domains within the individual modules. Finally, although the modules may represent functional units of the protein, our studies suggest that important interactions between the modules may be critical to understanding the structure and function of the ligand-binding domains.

## Methods

### Library screening, cDNA isolation and sequence analysis

Using low stringency hybridization methods, we previously isolated a partial length cDNA corresponding to the fourth module of *Xenopus *IRBP [40]. This cDNA, termed XenB1, corresponds to the fourth or C-terminal module of IRBP (see cDNA map in Figure 1). In the present study, XenB1 was used to screen a *Xenopus *whole body stage 45 (33) swimming tadpole cDNA library generously provided by Dr.Douglas W. DeSimone (34). This λ Zap II library (Stratagene, La Jolla, CA) was screened under high stringency conditions as described by Rajendran *et al*. (1996) [25]. The cDNA was sequenced in pBluescript by the dideoxy chain-termination method using synthetic oligonucleotides and Sequenase version 2.0 (U.S.B., Cleveland, OH).

Sequence analysis and database searches were carried out with the Wisconsin Sequence Analysis Package (GCG). The PileUp program of the GCG package was used to generate the alignments. The ends of the sequences were weighted as gaps to reflect the alignment of the junction between modules and the alignment of the stop codons. Alignments used translated cDNAs of published C-terminal modules from human [82], bovine [27], goldfish [26], and zebrafish [25] IRBPs. Phylogenetic distances between the various IRBP modules were calculated by the ProtPars and Fitch programs of the PHYLIP phylogenetic analysis program package.

### Expression of full-length Xenopus IRBP and its individual modules as thioredoxin fusion proteins

The solubility of proteins expressed in *E. coli *can be enhanced by the use of thioredoxin as a fusion partner [52,83,84]. We expressed the full-length IRBP as well as its individual modules as thioredoxin-fusion proteins using pTrxFus and pThioHis vectors (Invitrogen, SanDiego, CA). The pThioHis vector incorporates a histidine patch on the surface of the thioredoxin [85]. cDNAs corresponding to the full-length IRBP, module 1, and module 2 were expressed in pThioHis; modules 3 and 4 were expressed in pTrxFus. IRBP cDNAs were amplified from Xen10a (Figure 1) using the oligonucleotides primers shown in Table 1. The amplified cDNAs were subcloned into the SmaI/Sal I site of pTrxFus or into the Stu I/Sal I site of pThioHisA. The pThioHis-IRBP constructs code for an additional three amino acid residues (GDP) at the N-terminus. The pTrxFus-IRBP construct codes for an additional 8 amino acid residues (SYCSNRYG) at the C-terminus. The cDNA corresponding to the fourth module has been described [40] and was previously used to express that module as a polyhistidine fusion protein in the pRSET system [51]. The cDNA was excised from pRSET with Bam HIand Nhe I, which cut in the plasmid's multiple cloning region 5' to the insert and in the cDNA's 3'-untranslated region, respectively. The cDNA was ligated into the Bam HI/Xba I site of pTrxFus. The pTrxFus and pThioHis constructs were used to transform GI724 and GI698, and Top10 *E. coli *respectively (InVitrogen) [86]. The reading frames of all plasmid constructs were confirmed by DNA sequencing.

Pilot expression cultures confirmed the size of the recombinant protein and were used to optimize the temperature and duration of protein expression. The thioredoxin fusion proteins were released from the *E. coli *by subjecting one ml of a 5.0 OD_550 _culture resuspended in 20 mM Tris pH 8.0 2.5 mM EDTA to repeated sonication and flash freezing in liquid N_2 _[86]. The soluble and insoluble fractions were separated at 16,000 g and analyzed by SDS-(8–10%)PAGE. The optimal temperature and incubation times for each of the recombinant IRBPs is given in Table I.

Preparative fermentations were carried out in a 7-L reactor (Applikon, Foster City, CA). After the cells reached an OD_550 _of 0.5, the temperature was lowered and isopropyl β-D-thiogalactopyranoside was added to a concentration of 1 mM. The incubation was continued for 4 to 21 hrs depending on the protein being expressed (see Table 1). The cells were harvested by centrifugation and resuspended at 4°C in 50 mM Tris pH 7.4, 100 mM NaCl with 1 mM phenylmethysulfonyl-fluoride, 1.4 μM pepstatin A, 0.3 μM aprotinin, and 2 μM leupeptin as protease inhibitors. The bacteria were ruptured with a French pressure cell, and the insoluble and soluble fractions separated at 12,000 g for 30 min at 4°C and stored at -80°C.

For most fermentations of the individual modules, approximately 75–85% of the product was present in the soluble fraction. The exception was module 3 where only 15% of the product was expressed in a soluble form. For this module we found that 90% of that in the insoluble fraction could be recovered using a modification of a procedure commonly used for solubilization of glutathione *S*-transferase fusion proteins [87]. The crude pellet was resuspended in 5 mM DTT, 1% sarkosyl (N-dodecanoylsarcosinate), 100 mM NaCl, 1 mM EDTA, 10 mM Tris at pH 8.0. Triton X used in the original description of the method [87] was not necessary for the module 3-thioredoxin fusion protein.

The IRBP fusion proteins were purified from the soluble fraction by a combination of ammonium sulfate precipitation, ion exchange chromatography, and affinity chromatography. Precipitation trials showed that a final concentration of ammonium sulfate at 14% provides the best compromise between purification and total yield. Ion exchange chromatography was carried out using Macro-Prep High Q Support (Bio-Rad, Hercules, CA). The recombinant protein was eluted from the column using a 200 – 750 mM NaCl gradient in 10 mM Tris at pH 7.4. The final purification step consisted of arsenical-based or metal ion affinity chromatography. The former utilized immobilized phenylarsine oxide which binds the vicinal thiols of the thioredoxin active site(-Cys-Gly-Pro-Cys-) [88-90]. Agarose-4-aminophenylarsine oxide was activated with 20 mM β-mercaptoethanol (β-ME) and washed in lysis buffer containing 1 mM β-ME. The soluble *E. coli *fraction was incubated with gentle agitation for two hrs to overnight in a 15% slurry of the resin in the presence of 1 mM β-ME. The resin was washed in a column until the absorbance reached baseline. The protein was eluted with a 1 to 100 mM β-ME step or linear gradient. For metal ion affinity chromatography Chelating Sepharose Fast Flow resin was activated with Ni^2+ ^according to the suggested protocol of the manufacturer (Pharmacia Biotech, Uppsala, Sweden). The protein was eluted from the column using a 0 – 100 mM linear imidizole gradient. Protein binding and elution were carried in the presence of 900 mM NaCl to minimize nonspecific interactions with the resin. The purified protein was dialyzed against PBS (137 mM NaCl, 2.7 mM KCl, 1.4 mM KH_2_PO_4_, 1.4 mM sodium phosphate, pH 7.3) before being frozen as aliquots in liquid nitrogen (It was necessary to lower the salt concentration before freezing the protein to prevent precipitation upon thawing.). We found that one freeze/thaw cycle did not change the binding parameters as measured by fluorescence enhancement, or quenching of endogenous protein fluorescence or energy transfer. The frozen aliquots were held at -80°C until use. We only used protein that had been frozen and thawed no more than once.

The concentration of the purified IRBP in the stock frozen aliquots was determined by amino acid analysis and UV spectroscopy. The amino acid analysis was performed on a PICO-TAG system (Waters, Milford, MA) using phenylisothiocyanate derivatives [91]. An internal standard was included in each assay. For UV spectroscopy, extinction coefficients were calculated for each of the recombinant IRBPs from their amino acid sequence using a previously described method (Table 1) [92,93]. The concentration of the purified recombinant proteins determined by amino acid analysis was generally 10 – 25% less than that determined by UV spectroscopy. UV spectroscopy over estimates the concentration probably due to the presence of small amounts of nucleic acid or inaccuracies associated with the extinction coefficient calculation. For this reason we used the protein concentration value determined by amino acid analysis in our calculation of binding stoichiometry. The purity of the recombinant IRBPs was determined by laser densitometric analysis of Coomassie blue stained SDS 8–10% polyacrylamide gels (Molecular Dynamics, Sunnyvale, CA). The final purity of the recombinant IRBPs ranged from 90 to 99%.

### Liquid chromatography tandem mass spectrometry (LC-MS/MS)

Analysis of protein digests by LC-MS/MS has been recently reviewed [94]. Briefly, a minced Coomassie blue stained SDS polyacrylamide gel slice containing ~3 μg of purified protein was destained in 50% methanol, dehydrated in acetonitrile, reduced with 10 mM DTT/0.1 M ammonium bicarbonate at 55°C for 1 hr and alkylated in 50 mM iodoacetamide/0.1 M ammonium bicarbonate. The gel pieces were then washed in 0.1 μM ammonium bicarbonate, dehydrated in acetonitrile and dried. This was followed by rehydration in 12.5 ng/μl trypsin in 50 mM ammonium bicarbonate on ice for 45 min. Excess trypsin solution was removed and the digestion carried out in 50 mM ammonium bicarbonate overnight at 37°C. Peptides formed were extracted from the polyacrylamide in 50% acetonitrile/5% formic acid. The extracts were combined and evaporated to < 20 μl for LC-MS/MS analysis. The LC-MS/MS system consisted of a Finnigan-MAT TSQ7000 system with an electrospray ion source interfaced to a 10 cm × 75 μm internal diameter POROS 10 RC reversed phase capillary column. One μl volumes of the extract were injected and the peptides eluted by an acetonitrile/0.1 acetic acid gradient. The digest was analyzed by capillary LC-electrospray mass spectrometry to measure the molecular weight of the peptides present. Amino acid sequences of the detected peptides were determined by collisionally activated dissociation using LC-electrospray-tandem spectrometry with argon as the collision gas.

### Protection of all-trans retinol

Full-length *Xenopus *IRBP was evaluated for its ability to protect all-*trans *retinol from degradation as described by Crouch *et al*. (1992) [11]. Briefly the absorbance of all-*trans *retinol at 325 nm in the presence and absence of the recombinant protein was monitored as a function of time using a Hitachi U2000 spectrophotometer.

### Fluorometric titrations

Enhancement of retinol fluorescence and quenching of the intrinsic protein fluorescence were used to monitor binding of all-*trans *retinol to the recombinant IRBPs. We previously derived an equation that is generally useful for describing such quantum yield changes that are consequent upon ligand binding [51]. This equation has been previously applied to the analysis of binding by monitoring the enhancement of retinol fluorescence [50,95]. In this case, where excitation is at 330 nm and emission is monitored at 480, the fluorescence enhancement (*F*_*enh*_) depends on the total retinol concentration (*R*_*t*_), the dissociation constant (*K*_*d *_^*all-trans*^), the number of retinol-binding sites per protein molecule (*N*) and the total protein concentration (*P*_*t*_) [51].

Assuming that there is a single type of noninteracting site on the protein, (*F*_*enh*_) is expected to be linearly related to the concentration of bound retinol molecules. *F*_*enh *_is given by the relation

Fenh=C1+C2[Rtall−trans−(Rtall−trans+NPt+Kdall−trans)2−4NPtRtall−trans]
 MathType@MTEF@5@5@+=feaafiart1ev1aaatCvAUfKttLearuWrP9MDH5MBPbIqV92AaeXatLxBI9gBaebbnrfifHhDYfgasaacH8akY=wiFfYdH8Gipec8Eeeu0xXdbba9frFj0=OqFfea0dXdd9vqai=hGuQ8kuc9pgc9s8qqaq=dirpe0xb9q8qiLsFr0=vr0=vr0dc8meaabaqaciaacaGaaeqabaqabeGadaaakeaacqWGgbGrdaWgaaWcbaGaemyzauMaemOBa4MaemiAaGgabeaakiabg2da9iabdoeadnaaBaaaleaacqaIXaqmaeqaaOGaey4kaSIaem4qam0aaSbaaSqaaiabikdaYaqabaGcdaWadaqaaiabdkfasnaaDaaaleaacqWG0baDaeaacqWGHbqycqWGSbaBcqWGSbaBcqGHsislcqWG0baDcqWGYbGCcqWGHbqycqWGUbGBcqWGZbWCaaGccqGHsisldaGcaaqaamaabmaabaGaemOuai1aa0baaSqaaiabdsha0bqaaiabdggaHjabdYgaSjabdYgaSjabgkHiTiabdsha0jabdkhaYjabdggaHjabd6gaUjabdohaZbaakiabgUcaRiabd6eaojabdcfaqnaaBaaaleaacqWG0baDaeqaaOGaey4kaSIaem4saS0aa0baaSqaaiabdsgaKbqaaiabdggaHjabdYgaSjabdYgaSjabgkHiTiabdsha0jabdkhaYjabdggaHjabd6gaUjabdohaZbaaaOGaayjkaiaawMcaamaaCaaaleqabaGaeGOmaidaaOGaeyOeI0IaeGinaqJaemOta4Kaemiuaa1aaSbaaSqaaiabdsha0bqabaGccqWGsbGudaqhaaWcbaGaemiDaqhabaGaemyyaeMaemiBaWMaemiBaWMaeyOeI0IaemiDaqNaemOCaiNaemyyaeMaemOBa4Maem4CamhaaaqabaaakiaawUfacaGLDbaaaaa@8490@

where C_1 _and C_2 _are parameters that do not vary during our measurements and can be assumed to be constants [51]. The equation was fit to the data by nonlinear least-squares analysis. To follow the quenching of the protein's intrinsic fluorescence the excitation and emission wavelengths were set to 280 nm and 340 nm, respectively, while the protein was titrated with all-*trans *retinol. For the quenching studies the inner filter effect was accounted for by graphical correction as previously described [56]. Fluorescence measurements were made using an SLM 8000_TM _C photon counting spectrofluorometer corrected for wavelength dependence of source energy and detector response.

To describe the displacement of all-*trans *retinol by 11-*cis *retinaldehyde, Equation 1 was modified to take into account the competition between the two ligands. The apparent dissociation constants for all-*trans *retinol measured under these conditions is related to the dissociation constant of 11-*cis *retinaldehyde through the following expression.

Kapp=Kdall−trans[1+Rt11−cisKd11−cis]
 MathType@MTEF@5@5@+=feaafiart1ev1aaatCvAUfKttLearuWrP9MDH5MBPbIqV92AaeXatLxBI9gBaebbnrfifHhDYfgasaacH8akY=wiFfYdH8Gipec8Eeeu0xXdbba9frFj0=OqFfea0dXdd9vqai=hGuQ8kuc9pgc9s8qqaq=dirpe0xb9q8qiLsFr0=vr0=vr0dc8meaabaqaciaacaGaaeqabaqabeGadaaakeaacqWGlbWsdaWgaaWcbaGaemyyaeMaemiCaaNaemiCaahabeaakiabg2da9iabdUealnaaDaaaleaacqWGKbazaeaacqWGHbqycqWGSbaBcqWGSbaBcqGHsislcqWG0baDcqWGYbGCcqWGHbqycqWGUbGBcqWGZbWCaaGcdaWadaqaaiabigdaXiabgUcaRmaalaaabaGaemOuai1aa0baaSqaaiabdsha0bqaaiabigdaXiabigdaXiabgkHiTiabdogaJjabdMgaPjabdohaZbaaaOqaaiabdUealnaaDaaaleaacqWGKbazaeaacqaIXaqmcqaIXaqmcqGHsislcqWGJbWycqWGPbqAcqWGZbWCaaaaaaGccaGLBbGaayzxaaaaaa@58DE@

The origin of the above expression is discussed in reviews dealing with competition assays [57]. Substitution of equation 2 into equation 1 yields the following expression.

Fenh=C1+C2[Rtall−trans−(Rtall−trans+NPt+Kdall−trans+Kdall−transRt11−cisKd11−cis)2−4NPtRtall−trans]
 MathType@MTEF@5@5@+=feaafiart1ev1aaatCvAUfKttLearuWrP9MDH5MBPbIqV92AaeXatLxBI9gBaebbnrfifHhDYfgasaacH8akY=wiFfYdH8Gipec8Eeeu0xXdbba9frFj0=OqFfea0dXdd9vqai=hGuQ8kuc9pgc9s8qqaq=dirpe0xb9q8qiLsFr0=vr0=vr0dc8meaabaqaciaacaGaaeqabaqabeGadaaakeaacqWGgbGrdaWgaaWcbaGaemyzauMaemOBa4MaemiAaGgabeaakiabg2da9iabdoeadnaaBaaaleaacqaIXaqmaeqaaOGaey4kaSIaem4qam0aaSbaaSqaaiabikdaYaqabaGcdaWadaqaaiabdkfasnaaDaaaleaacqWG0baDaeaacqWGHbqycqWGSbaBcqWGSbaBcqGHsislcqWG0baDcqWGYbGCcqWGHbqycqWGUbGBcqWGZbWCaaGccqGHsisldaGcaaqaamaabmaabaGaemOuai1aa0baaSqaaiabdsha0bqaaiabdggaHjabdYgaSjabdYgaSjabgkHiTiabdsha0jabdkhaYjabdggaHjabd6gaUjabdohaZbaakiabgUcaRiabd6eaojabdcfaqnaaBaaaleaacqWG0baDaeqaaOGaey4kaSIaem4saS0aa0baaSqaaiabdsgaKbqaaiabdggaHjabdYgaSjabdYgaSjabgkHiTiabdsha0jabdkhaYjabdggaHjabd6gaUjabdohaZbaakiabgUcaRmaalaaabaGaem4saS0aa0baaSqaaiabdsgaKbqaaiabdggaHjabdYgaSjabdYgaSjabgkHiTiabdsha0jabdkhaYjabdggaHjabd6gaUjabdohaZbaakiabdkfasnaaDaaaleaacqWG0baDaeaacqaIXaqmcqaIXaqmcqGHsislcqWGJbWycqWGPbqAcqWGZbWCaaaakeaacqWGlbWsdaqhaaWcbaGaemizaqgabaGaeGymaeJaeGymaeJaeyOeI0Iaem4yamMaemyAaKMaem4CamhaaaaaaOGaayjkaiaawMcaamaaCaaaleqabaGaeGOmaidaaOGaeyOeI0IaeGinaqJaemOta4Kaemiuaa1aaSbaaSqaaiabdsha0bqabaGccqWGsbGudaqhaaWcbaGaemiDaqhabaGaemyyaeMaemiBaWMaemiBaWMaeyOeI0IaemiDaqNaemOCaiNaemyyaeMaemOBa4Maem4CamhaaaqabaaakiaawUfacaGLDbaaaaa@A768@

### Computational ligand docking and homology modeling

The Molecular Operating Environment (MOE 2005.08; Chemical Computing Group, Montreal, Canada) software package running on a G5 dual 2.7 GHz PowerPC workstation was used for the ligand docking and homology modeling work. The *site finder *option of MOE that automatically identifies internal cavities within a receptor protein was used to locate possible ligand-binding sites in the crystal structure of *Xenopus *IRBP functional unit 2 (X2) (pdb code: 1J7X) [39]. An *all-trans *retinol molecule was chosen as the ligand. Default parameters including partial charges on amino acids and the ligand were turned on, and full conformational searches for the ligand was carried out. The pose with the best score was selected as the model for the docked ligand. To account for conformational flexibility of the active site pocket, the immediate neighborhood of the retinol-binding site (within 4.5 Å of the ligand) was energy minimized by restrained minimization.

Atomic models of modules 1, 3 and 4 of *Xenopus *IRBP (X1, X3 and X4, respectively) were built based on their sequence homologies with X2 utilizing its crystal structure and the homology modeling method implemented in MOE. In this method, an intermediate model of the target protein molecule is built using a Boltzmann-weighted randomized modeling procedure [96], combined with specialized logic for the proper handling of insertions and deletions [97]. Backbone coordinates for the sequences are built by searching high-resolution structures from the Protein Data Bank (PDB). The side chain coordinates for the non-conserved/insertion regions are obtained from an extensive rotomer library generated by systematic clustering of high-resolution PDB data. For modeling loops, a contact energy function is calculated for each candidate that is weighed by Boltzmann function to derive the coordinates. The best intermediate model thus obtained is energy minimized to remove bad van der Waals contacts. The final model is then generated either as the average of the atom coordinates of the intermediate models, or as the coordinates of the intermediate model that scored best according to the packing quality function. PyMOL was used for analysis and illustration purposes.

## Abbreviations

Carboxy-terminal processing protease (CtpA); dithiothreitol (DTT); β-mercaptoethanol (β-ME); isopropylthio-β-D-galactoside (IPTG); interphotoreceptor retinoid-binding protein (IRBP).

## Authors' contributions

FGF conceived the study, carried out the molecular genetic studies, participated in the sequence alignment, and drafted the manuscript. CAB participated in the design of the study, and performed the biochemical experiments. DG performed the structural modeling and docking studies. All authors read and approved the final manuscript.
